# Draft Genome Sequence of Simplicillium aogashimaense 72-15.1, a Putative Endophyte of Brachiaria brizantha

**DOI:** 10.1128/MRA.00366-20

**Published:** 2020-07-02

**Authors:** Ruy Jauregui, Linda J. Johnson, Suliana E. Teasdale

**Affiliations:** a AgResearch Limited, Grasslands Research Centre, Palmerston North, New Zealand; University of California, Riverside

## Abstract

Here, we report a 29-Mb draft genome sequence of strain 72-15.1 of Simplicillium aogashimaense (Cordycipitaceae, Hypocreales). Strain 72-15.1 was a unique fungal isolate obtained from asymptomatic tillers of the tropical grass Brachiaria brizantha.

## ANNOUNCEMENT

Simplicillium aogashimaense strain 72-15.1 is a putative fungal endophyte of Brachiaria brizantha (host accession number CIAT 16320) and was isolated from an asymptomatic leaf (1). This species was originally described from soil samples in Asia (2). Preliminary identification of *S. aogashimaense* strain 72-15.1 was based on morphological characteristics and a phylogenetic analysis with the internal transcribed spacer (ITS) region (1). *S. aogashimaense* strain 72-15.1 was isolated only once from *B. brizantha* and was the only species of the *Simplicillium* genus (as described by Zare and Gams [3]) to be isolated from nine species of *Brachiaria* grasses (1).

The 29-Mb draft genome of *S. aogashimaense* strain 72-15.1 is 1 of only 13 genomes of *Simplicillium* species to have been described thus far (4, 5). *Simplicillium* species are both ecologically and economically interesting due to their widespread distribution, diverse host specificities, varied lifestyles (4, 5), utility as biocontrol agents (6–8), and production of bioactive compounds (9–13). Previously, we showed high antifungal activity of the sequenced strain 72-15.1 against the phytopathogenic fungi Alternaria alternatum, *Bipolaris* sp. aff. *sorokiniana*, and Curvularia trifolii (1). The genome of strain 72-15.1 can therefore be mined for the discovery of these antifungal secondary metabolites, as well as for other metabolites that may be responsible for the antimicrobial properties exhibited by some members of this genus against bacterial and fungal plant pathogens as well as plant parasitic nematodes (8, 14, 15).

To obtain fungal material for DNA extraction, a pure culture was lacerated with sterile water, and the resulting solution was spread onto a sheet of cellophane on the surface of a peptone-dextrose agar (PDA) plate and then incubated at 24°C for ∼4 days. High-quality DNA was extracted from strain 72-15.1 using a Zymo Research Quick-DNA fungal/bacterial miniprep kit, following the manufacturer’s protocol, except mycelium was ground with a plastic pestle fitted to a drill under liquid nitrogen. Two libraries were constructed for whole-genome shotgun sequencing, one for paired-end reads with an insert size of 350 nucleotides (nt) and one for mate pair reads with an insert size of 7 kb, using the Illumina TruSeq DNA Nano low-throughput (LT) 350-bp and Illumina Nextera mate pair gel-plus kits. The libraries were sequenced on an Illumina HiSeq 2000 instrument using v3 chemistry. The instrument produced 30 million paired-end read pairs and 33 million mate pair read pairs with a read length of 125 nt. All paired reads were quality trimmed and filtered using Trimmomatic v0.33 (16) and assembled using Edena v3.2 (17) with default parameters. Contigs shorter than 200 nt were discarded. The contigs were further assembled into scaffolds using the program SSPACE v3.0 (18) with the parameters -k 5, -a 0.7, -x 1, -m 30, and -o 20. The pipeline produced 22 scaffolds with a final genome size of 29.247 Mb, an *N*_50_ value of 4 Mb, and a 49% GC content. A BUSCO (19) run using the Ascomycota database vodb9 (20) and Aspergillus nidulans as the gene predictor template reported a genome completeness of 99.1%, 5 duplicated benchmarking universal single-copy orthologs (BUSCOs) (0.4%), and 5 fragmented BUSCOs (0.4%).

### Data availability.

This whole-genome shotgun project has been deposited at DDBJ/ENA/GenBank under the accession number JAALXG000000000. The version described in this paper is version JAALXG010000000. The raw Illumina data from BioProject PRJNA599221 were submitted to the NCBI Sequence Read Archive (SRA) under accession numbers SRX7508659 and SRX7508658.
